# A new dawn for critical care research

**DOI:** 10.1186/2197-425X-1-1

**Published:** 2013-10-29

**Authors:** Mervyn Singer, Elie Azoulay, Jean-Daniel Chiche

**Affiliations:** Bloomsbury Institute of Intensive Care Medicine, University College London, Cruciform Building, Gower Street, London, WC1E 6BT UK; Medical ICU, Hôpital Saint Louis, 1 Avenue Claude Vellefaux, Paris, 75010 France; Hôpital Cochin, 27, rue du Faubourg Saint-Jacques, Paris, 75014 France

## Editorial

Critical care is a rapidly growing specialty. As a mark of its increasing maturity and health, its practitioners are increasingly challenging long-held dogma and revising practice guidelines. Critical care certainly punches well beyond its weight, accounting for a disproportionately large number of clinical trials reported in the highest impact factor journals. An impressive level of altruistic collaboration has often driven such studies, even on shoestring budgets. Outcomes have improved, in large part due to improvements in general care, early recognition of deterioration, and avoidance of iatrogenic harm.

Notwithstanding these achievements, few novel drugs or other interventions that have impacted positively on patient outcomes have come to the fore over the last 20 to 30 years. This disconnect highlights the crucial disparity between the clinical and biological phenotypes of critical illness. Treatment strategies based on clinical syndromic presentations, e.g. for sepsis, acute respiratory distress syndrome, and acute kidney injury, have repeatedly fallen short of their initial promise. The heterogeneity of the population with respect to age, gender, underlying comorbidity, trajectory and severity of illness, time to presentation, infecting organism, and the type and number of affected organs adds further complexity to trialing within an already challenging patient cohort. Some interventions, albeit based on a seemingly sound rationale, have even been associated with patient harm.

It is starkly obvious that improving our still limited grasp of the underlying pathophysiology is key to making major advances in patient management. Yet, basic/translational research has been traditionally relegated to a minor role and is generally undervalued by the clinical community. This is reflected by a relatively low profile at (inter)national congresses, fewer funding opportunities, and comparatively few basic scientists specifically focusing on critical care conditions and pathologies. Even the specialist journals generally eschew basic/translational papers, particularly those involving animal or laboratory models. However, in their defence, the quality of such work is somewhat variable, thus creating a perfect Catch 22 situation of struggling to attract the funding and quality researchers needed to improve the overall calibre of the research output.

With this in mind, *Intensive Care Medicine*, the official journal of the European Society of Intensive Care Medicine (ESICM), has taken the bold step of splitting into two separate but closely linked sister journals. *Intensive Care Medicine* will continue as a solely clinical journal, reporting clinical trials, process of care, ethical issues, health economics, and so forth. *Intensive Care Medicine Experimental* (*ICMx*) will focus on experimental research, stretching from cell and *in silico* models, through *in vivo* and *ex vivo* laboratory studies, to human volunteer and patient studies, where the emphasis is on biology rather than on clinical outcomes.

The risk is that clinicians will choose to ignore this stand-alone offering. Yet, we hope that offering a dedicated platform for such research will promote its importance, elevate standards, improve transferability of findings from bench to bedside (and back), and stimulate clinicians, basic scientists and, particularly, the next generations of researchers to actively participate. In conjunction, other initiatives will be taken by ESICM to elevate basic and translational research and encourage engagement.

*Intensive Care Medicine Experimental* (icm-experimental.com) will be an online-only electronic journal that, by being open access, will guarantee rapid and widespread dissemination of data, but with the assurance of decent, high-quality peer review [1]. Being part of the SpringerOpen stable, it provides quality processing of manuscripts and numerous portals to publicize the papers (including social media). This will be complemented by the ESICM website that will promote the journal on its front page. A strong endorsement has come from the ready acceptance by a cadre of 40 top-flight researchers to take on active roles as Senior Editors and Editors, with commitments to lend their significant expertise to the peer review process, to submit their research, and to offer enthusiastic promotion in support of this fledgling journal. These researchers are a healthy mix of basic scientists and clinician scientists whose research activities have led them to embrace experimental research in distinct areas of critical care (inflammation, immunity, respiratory, metabolic, neurological, endocrine, trauma, and so forth). *ICMx* will thus, uniquely, be driven by the critical care community with a sole focus on basic to translational research.

As with any new offering, the first few years will be challenging. We are, however, quietly confident that it will succeed and sincerely hope the scientific community will join in this exciting journey.
